# Reablement in a small municipality, a survival analysis

**DOI:** 10.1186/s12913-021-06910-6

**Published:** 2021-08-26

**Authors:** Kjartan Sarheim Anthun, Monica Lillefjell, Kirsti Sarheim Anthun

**Affiliations:** 1 Department of Health Research, SINTEF Digital, Trondheim, Norway; 2 Department of Public Health and Nursing, Norwegian University of Science and Technology, Trondheim, Norway; 3 Department of Neuromedicine and Movement Science, Norwegian University of Science and Technology, Trondheim, Norway

**Keywords:** Reablement, Home care, Municipality, Restorative homecare, Health care

## Abstract

**Background:**

Reablement is a municipal service given to patients at home. The purpose of the service is to assist recovery after hospital discharges or other sudden changes in a patient’s functional level. The service is often provided by a team of nurses, physiotherapists, and occupational therapists. The purpose of this paper is to compare users of this service to users who receive traditional home care services. Outcomes to be measured are risk of long-term care and mortality.

**Methods:**

All users of health and care services in a Norwegian municipality were eligible for inclusion. Data was extracted from the local user administrative database. Users were divided in two groups: those who received reablement and those home care users who did not receive reablement service. Propensity score matching was used to match users based on age, sex, and level of functioning in activities of daily living (ADL). Survival analysis was deployed to test if the reablement users had different risk of becoming long-term care users, and whether the mortality rate differed for this group.

**Results:**

153 reablement users were included in the study. These were matched to 153 non-reablement home care users. The groups had similar distributions of age, sex, and level of functioning when starting their service trajectories. Regressions showed that reablement users had lower risk of using long-term care services in the study period (time at risk up to 4 years), and lower mortality. However, none of these estimates were statistically significant.

**Conclusions:**

The study indicates that the reablement users in one municipality had lower use of long -term care and lower mortality when properly estimated, but numbers were too small for statistical significance to be found.

**Supplementary Information:**

The online version contains supplementary material available at 10.1186/s12913-021-06910-6.

## Background

The rapidly ageing population is putting substantial pressure on health care systems in the 21st century [1]. Norway has a universal health and social care system; all citizens have universal access to publicly funded free services, though some specific services require only a small out-of-pocket payment. The Norwegian system is a multi-level model with state owned- and -funded hospitals responsible for medical interventions that cannot be performed in the community, whereas municipalities are assigned responsibility for primary health care and for long-term care such as institutional care (i.e., nursing homes and similar) and home-based care [2]. Due to the last few decades of new legal obligations and technical and medical advances, municipalities have been given increasing responsibilities for health care [3, 4]. Along with this, municipalities are expected to pay more attention to cost control and find smarter ways to organize services [5].

Historically, Norway has spent more on long-term care than other European countries [6]. The demand for home care, long-term care and specialized care are expected to increase in the coming years as the number of people with long-term conditions rise. Home care is usually provided to a much larger part of the population than more costly care such as long-term institutional care and specialized care in hospitals. However, the profile and mix of home care and long-term services offered by municipalities vary greatly as municipalities to a large extent are free to organize their services in the way they find adequate [7, 8].

To manage the demands on care services, municipalities look for new, innovative, and cost-effective solutions to providing health care services. One such solution has been the introduction of reablement services (also called *restorative homecare* in the literature)^1^. Reablement is a time-limited home care service provided by a multi-disciplinary team often consisting of a nurse, physiotherapist, and occupational therapist. The service is a person-centred and goal-directed rehabilitation intervention where the aim is to maintain and improve the functional independence of older adults. It is a service provided to assist recovery after hospital discharge or other sudden change in functional level. Facilitating participation and activity among older adults and enabling mastery of activities of daily living are core principles in reablement services [9–11]. Reablement services have been assumed to be cost effective [12]; as the service aim to contribute to supporting the elderly to being as self-sufficient as possible and hence rely less other home care services.

The very definition of reablement varies [13, 14], hence also the outcomes. Outcomes assessed in the literature range from cost savings [10] to improvements in various clinical outcomes; i.e. functional abilities and strength [15–17], execution of activities of daily living (ADL) [18], gait speed [19], social support [20], loneliness [21], and a better quality of life for the patient [21, 22].

A large part of the reablement research in Norway is of a qualitative nature [20, 23], however there are examples of quantitative studies [24, 25]. Globally there are very few high-quality quantitative effect studies. A Cochrane review [26] presented only two studies and claimed that the evidence had “very low quality” and that there was “[…] considerable uncertainty regarding the effects of reablement” [26]. More quantitative studies with a long time span are wanted [26].

The present study aims to evaluate health effects of reablement service over a long time span. The purpose is to quantitatively compare patients receiving reablement services with patients who receive traditional home care services. This is done using data from one municipality. The data had a long time span, some users had a follow up time of up to 4 years. We test two outcome measures: risk of long-term care and mortality.

## Methods

The present study is a retrospective observational study that aims to evaluate health effects of reablement service in a municipality.

### Description of the data

The context of this study is a relatively small, suburban municipality in Norway with less than 15 000 inhabitants. Reablement was implemented into home-care services seven years prior to this study.

Data isolated to the reablement service was not available from the national registries on health care services in Norway. To collect data, we had to extract data directly from the patient administrative system of the municipality included in this study. The system contained all users that had applied for, or received, municipal health or care services in that municipality in the period 1993 to 2018. From this we extracted the history of use of services and individual needs for care. Need for care is measured for each user that applies for municipal health and care services, on activities of daily living functional scales. These are 15 specific scales are which are nationally standardized and include both physical (PADL) and instrumental activities of daily living (IADL) [27].

The data collected contained 4 872 individuals, however this included also test data, deceased individuals, and people who had moved from the municipality. Thus, the real number of health and care users in the municipality was less than 1000.

**Fig. 1:**
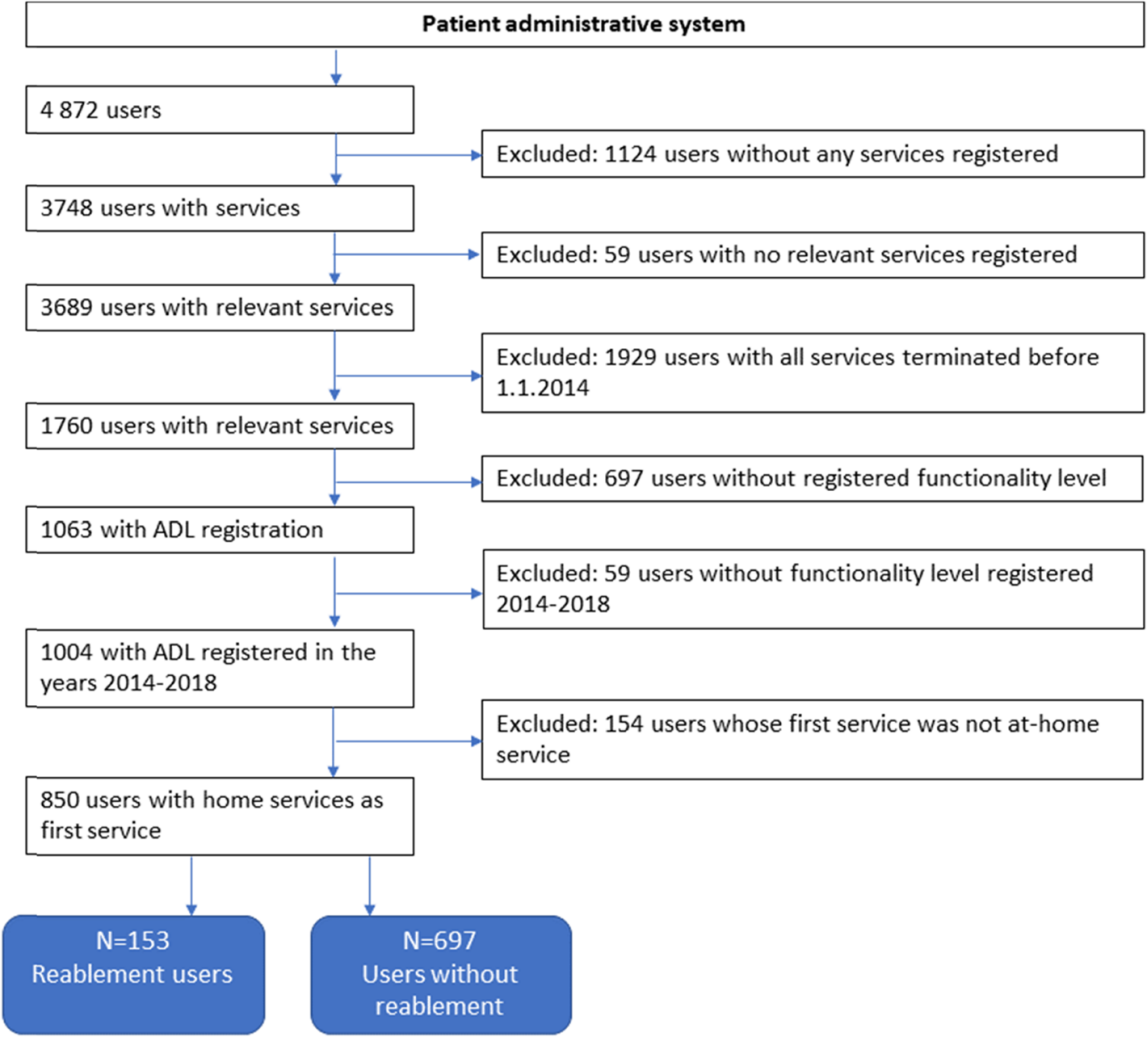
Data inclusion/exclusion flow chart

To select the reablement users and the control group some users where excluded. These were users without any services registered, users with no relevant services registered (i.e. parking permissions), users with services stopped prior to 1.1.2014, users without registered need for care (ADL measurement), users without need for care (ADL measurement) within the time frame of the study, and finally we also excluded users whose first service in the municipality was not an at-home service. See Fig. 1 for inclusion and exclusion flow chart. This data washing resulted in 153 reablement users and 697 without reablement.

Functionality level is measured as a mean across 15 activities of daily living (both PADL and IADL). Each activity is measured on non-linear 1–5 scale where 1 indicates no problem/challenge, 2 no need for assistance, 3 some needs for assistance, 4 large needs for assistance and 5 indicates complete dependence.

### Methods

#### Survival analysis

The purpose of survival analysis is to analyse the possibility of an unwanted event by comparing different groups under risk. The main outcome variable in survival analysis is time until an event occurs [28], typically referred to as *survival time*. The unwanted event can be any number of different outcomes that may happen to an individual.

Our study examines two outcomes: mortality and long-term care. Both outcomes are operationalized in two ways depending on the type of analysis.

Date of death was registered in the patient administrative system for the diseased. For the survival analyses we measured time to death as the number of days from the first registered service to death. In the regressions comparing the reablement and non-reablement groups, the outcome is operationalized as a dummy, i.e., death or survival.

Long-term institutional care is a level of service for users in the municipality. This will typically include nursing homes. The outcome in our analyses was operationalised twofold: (1) as a dummy (yes/no) if the user receives long term care at any point, and (2) the number of days from the first registered service to the possible unwanted outcome.

In the analysis we will use two main methods. The first is to produce Kaplan-Meier curves. These curves display the cumulative probability of survival at any given point in time after the start of the study. Survival is not limited only to avoiding death, but also avoiding any other predefined unwanted event. In the present study we do look at death and examine the use of long-term care as unwanted outcomes, and survival is interpreted as avoiding these outcomes over time. The Kaplan-Meier curves will indicate if the unwanted event occurs sooner or later for the different groups. The best possible outcome as time passes (i.e. towards the right-hand side of each graph) is for each group to have a large share surviving – i.e. not showing a declining curve. The Kaplan-Meier graph will then show two important traits; (1) what the end result is, that is; which group has the highest level at the end of the curve i.e. the longest time before an unwanted event occurs, and (2) how the development over time is for each group. We perform log-rank test to test if survivor functions of the two groups are equal.

We will also estimate Cox proportional hazard model. This is a mathematical method for analysing survival data. The method includes multivariate regressions to describe hazard ratios of the different variables, estimated using Maximum likelihood. Hazard is the potential for the unwanted event to occur, given the survival time. Cox proportional hazard models are reliable and robust for analysing survival data [28].

#### Propensity score matching

In this paper we test the outcome of one group of patients: reablement users. This is one specific service in one specific municipality. If we compare just the average of this service to the average of those that do not receive this service, we are likely to find differences due to selection bias. In our setting, it was not possible to achieve randomization. To overcome this, we employed a matching strategy.

For each user of reablement, we matched a home care user who did not receive reablement services. Propensity score matching [29] was the method applied to obtain the best possible match. By comparing similar age, sex, and level of ADL functioning, the method constructs as close a match as possible, so that the starting point for comparison is not all that different. Technically, propensity score matching involves a logistic regression to calculate the probability of a unit belonging to the treatment group (reablement) based on the included control variables (age, sex, and functionality level). The results after the matching were a set of weights that balanced the dataset so that the treatment and non-treatment groups can be matched and compared.

In the matching process we also estimated the average treatment effect which is the average difference between the reablement and regular home care users conditioning on the covariates (age, sex, and functionality level).

After matching we performed a simple OLS regression to test if there were differences between the reablement and non-reablement groups, even after both matching and controlling for age, sex, and functionality level.

## Results

After the selection process, 153 reablement users were included. Also included in the study were 697 non-reablement home care users.

**Table 1:** T-test of differences of means between non-reablement users prior to weighting/matching

Characteristic and outcome	Non-reablement users prior to weighting	Reablement users	Welch-T-test of difference in groups (t)	Two-sided T-test of difference in groups (p)
Age	55,83	73,512	-9,439	0,000
Share female	0,554	0,660	-2,485	0,014
Functionality level	1,662	1,656	0,157	0,876
Outcome: Long-term care	0,109	0,111	-0,074	0,941
Outcome: Mortality	0,196	0,196	0,013	0,989
N	697	153		

Table 1 shows that there are large demographical variations. The reablement users are significantly older than the non-reablement users. Related to this we also observe a difference in the gender balance in each group. The gender unbalance is most likely due to the age difference. To test the difference between these groups we perform a T-test of differences in mean. However, since the two groups are of different size and with difference variance, we apply a Welch T-test [30] using the Welch-Satterthwaite equation to estimate the pooled degrees of freedom [30, 31]. Regardless of the demographic variations we observe no distinction with regards to activities of daily living (user needs, or functionality level), long-term care or mortality.

Log-rank test of the data showed that we cannot differentiate between the survivor functions of the two groups.

### Matching

We saw in Table 1 that the reablement users were older, so to better compare outcomes we matched to create two more comparable groups. The 697 non-reablement home care users were weighted after propensity score matching to a total of 153 users to be comparable with the reablement group. Matching was done controlling for age, sex, and level of ADL functioning. Average treatment effects regression on the long-term care outcome was used to estimate weight. A similar Welch T-test showed that there were no significant differences with respect to the patient characteristics (age, share female and functionality level), however the differences in outcomes were larger but still not statistically significant. See Table 2 where it is shown that the groups of reablement users and non-reablement users are not that different, hence they have been properly matched.

**Table 2:** T-test of differences of means between non-reablement users after weighting/matching

Characteristic and outcome	Non-reablement users after matching	Reablement users	Welch T-test of difference in groups (t)	Two-sided T-test of difference in groups (p)
Age	72,858	73,512	-0,294	0,769
Share female	0,719	0,660	1,111	0,268
Functionality level	1,634	1,656	-0,358	0,721
Outcome: Long-term care	0,170	0,111	1,481	0,140
Outcome: Mortality	0,268	0,196	1,490	0,137
N	153	153		

**Fig. 2:**
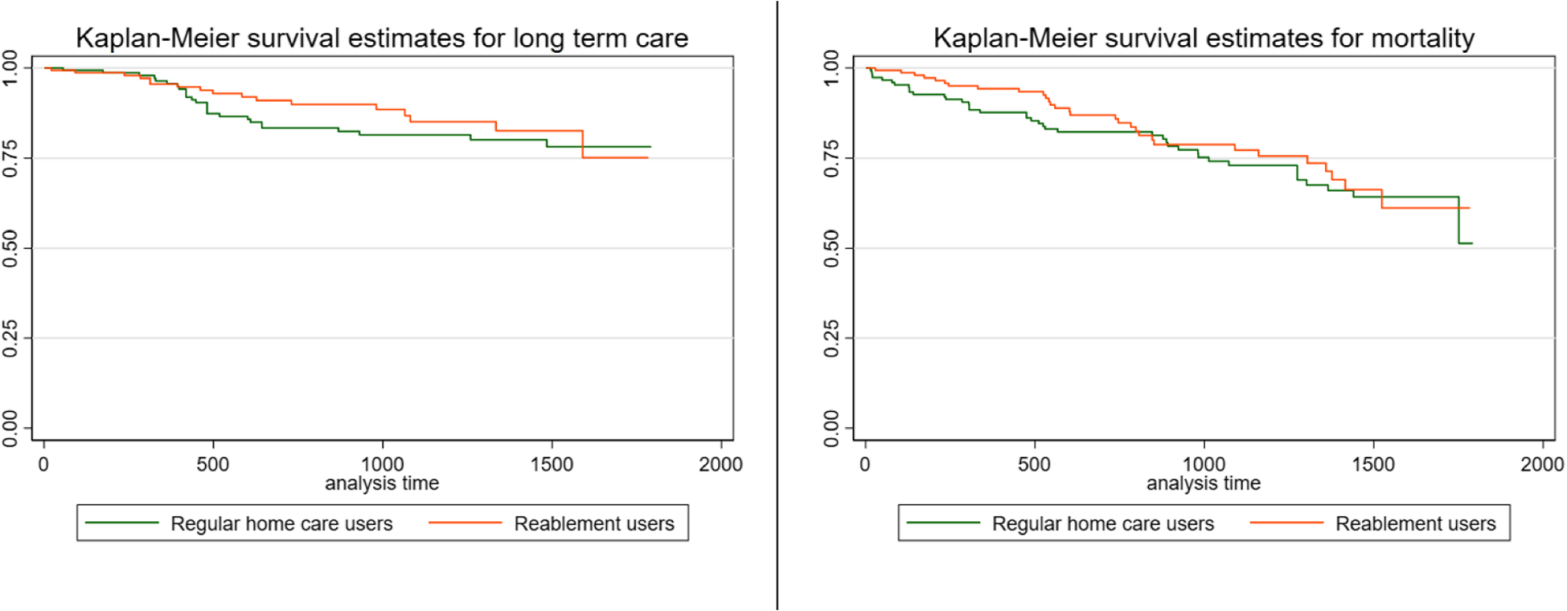
Kaplan-Meier survival estimates of long-term care and mortality, for non-reablement users and reablement users, matched. Share of each group surviving (y-axis) after days since first in need of services (x-axis)

Figure 2 shows Kaplan-Meier survival estimates after matching. Overall, the reablement users show a better survival in the first 1500 days for both outcomes

### Regression results

We performed several regressions to test if the reablement users had different outcomes than the non-reablement users.

As part of the survival analysis, we tested a cox proportional hazard model to see if there is an association regarding survival time in the two groups. This model was applied to the pooled data, i.e. 850 patients. The result from this analysis is a hazard ratio which compares the probability of unwanted events in the two groups. If the hazard ratio is above 1, the number of unwanted events is higher in the reablement group than in the non-reablement group. If the hazard ratio is 1, there is no difference in the number of unwanted events, and consequently if the hazard ratio is below 1, there are less unwanted events in the reablement group.

After the survival analysis, we also calculated average treatment effects on the pooled data. This measures the difference in the mean outcome by the reablement users and the non-reablement users. The estimation was done when calculating the matching weights in the propensity score matching. And finally, after matching, we performed a regular (ordinary least squares) regression on the weighted data to test the association and effect of reablement users on the outcomes. Table 3 below summarizes the results, and tables for cox proportional hazard model and matched regression are detailed in the additional files (see Additional file 1). In all regressions, both outcome measures were negative (or had/showed hazard ratio below 1) after controlling for age, sex, and functionality level. The P value from the regressions is the probability that the coefficient is different from 0 in the linear regression, or different from 1 in the hazard model.

**Table 3:** Summary of regressions; for cox proportional hazard model hazard ratio and P-value for dummy variable for reablement users, average treatment effect, and from linear regression, coefficient, and p-value for dummy for reablement users

	Long-term care	(p)	Mortality	(p)
Pooled data, *N* = 850				
Cox proportional hazard model (hazard ratio)	0,775	0,356	0,702	0,087
Average treatment effect (propensity score matching estimator)	-0,054	0,013	-0,085	0,001
Matched data, *N* = 306 (OLS coefficient)	-0,057	0,132	-0,081	0,063

## Discussion

Important motivations for implementing reablement services are reduction in long-term care expenditure [10, 23], and the need to promote healthy ageing and participation [5, 26]. Reablement has spread rapidly across Norway during the last eight years [32]. However, the evidence on which factors predict better outcomes and effects is still lacking [26, 32]. Reducing the need for hospitalized care and long-term care, has been an important strategy for most national health care systems. The recent COVID-19 pandemic has accentuated this need. Reinforcing home based care and utilizing reablement services can be an important strategy in improving the sustainability of the whole health care system.

In this study we look at reablement service receivers and traditional home care service receivers in a small Norwegian municipality. Outcomes measured were risk of long-term care and mortality. We found that older adults receiving reablement seem to face increased risk of mortality and they were more likely to end up in long-term care. This was witnessed by the Kaplan-Meier graphs presented. The pattern was similar for both groups for the first 1000 days, but after 1000 days there was a slight divergence with the reablement users where the hazard estimates indicated that they had lower survival rate and a higher risk of both needing long-term care and mortality. However, these graphs do not control for age, sex or level of ADL functioning, and the reablement users were of a much higher age when they started their service in the municipality, thus there are natural causes for increased mortality in this group as time passes.

We counteracted this by controlling for age, sex, and functionality level. Subsequently, the effect changed and reablement users seemed to face reduced risk; both showing less use of long-term care and reduced mortality. However, this effect was not significant as the estimate of the confidence interval was large. Significance levels do largely depend on the number of users included. This study only included 153 reablement users, so small effects are not likely to be classified as significance. If the sample had been twice as large the effect would likely be classified as significant at the 0,05 level. We will argue that there is an effect of reablement, but the setting is too small to measure. Further research should be done on a larger scale to quantify effects of such services.

In addition to small number of included users, the study has three other limitations, all of them related. First, in gauging the effects of specific services, randomized controlled trials are still considered the gold standard. Allocation concealment is known to increase the effect size, and double-blinding participants is one way to resolve these issues [33]. However, such a study design is very difficult to implement as one must randomize at different levels; which municipalities get to offer this service, and within each municipality which users will receive this service. Both are unlikely to be easily implemented. Secondly, the effects here may not be the result of the service per se, but a specific effect of the included municipality. When studying only within one municipality, we cannot control for the threshold for inclusion or exclusion of this service imposed on the users by the municipality. In some municipalities reablement may be offered routinely to all new home care users, while other municipalities may only offer the service for users with specific rehabilitation needs. And the services provided by a reablement team may vary between municipalities, both by the length of service, and the intensity of the provided service. Furthermore, when studying in only one municipality, budgetary prioritization between services cannot be controlled for.

And finally, even though we have included a control group through matching, there may still be a quite strong selection bias. The ones that received reablement in this specific municipality were those that the municipality deemed to be most benefiting from this type of service. Selection into the reablement group also leaves a residual selection bias for those *not* receiving reablement services as they may be deemed *not* to benefit from such a service. These inherent biases are difficult to avoid. Since the number of included patients was low, it was not possible to study different effects within this patient group. Conclusions about effects must be taken with a caution since the patients were not randomly assigned the reablement service, but selected based on expected outcomes. To address and tackle the most important of these limitations, future studies should include more patients and several municipalities. This could provide significant evidence of the effects of reablement.

## Conclusions

This study demonstrated that reablement users in one municipality had lower use of long-term care and lower mortality when properly estimated, but due to low number of users the results were not significant. The strength of this paper is to properly match and estimate these effects. However, the study only included 153 reablement users, all from the same municipality. Implication of well managed reablement services is the possibility to save both costs and increase even life expectancy.

## Supplementary Information

**Additional file 1.** Supplementary file with regression tables. Cox proportional hazard model for long term care

## Data Availability

The data that support the findings of this study are available from the municipality of the study, but restrictions apply to the availability of these data, which were used under license for the current study, and so are not publicly available. Aggregated data are however available from the authors upon reasonable request and with permission of the municipality of the study.

## Footnotes

1 Commonly called *hverdagsrehabilitering* (everyday rehabilitation) or *hverdagsmestring* (everyday mastering) in Norwegian.
