# Supplementary material for: Reablement in a small municipality, a survival analysis
Source: BMC Health Serv Res. 2021 Aug 26;21:877. doi: 10.1186/s12913-021-06910-6 (PMC8390107; doi:10.1186/s12913-021-06910-6)
Supplement: Supplementary file 1 — Additional file 1. Supplementary file with regression tables. Cox proportional hazard model for long term care [file 12913_2021_6910_MOESM1_ESM.doc]

# Reablement in a small municipality, a survival analysis

Anthun, Kjartan Sarheim ^1^,^2^ [Corresponding author; kjartan.s.anthun@sintef.no]

Lillefjell, Monica ^3^

Anthun, Kirsti Sarheim ^1^,^3^

1: SINTEF Digital, department of Health Research, Norway

2: Department of Public Health and Nursing, Norwegian University of Science and Technology, Norway

3: Department of Neuromedicine and Movement Science, Norway

# Cox proportional hazard model for long term care

Number of subjects: 849

Number of failures: 93

Time at risk: 842 539

LR chi^2: 129,28

Log likelihood: -532,19

| **Variable** | **Hazard ratio** | **Std.error** | **Z score** | **P>|z|** | **95% confidence interval** |
| --- | --- | --- | --- | --- | --- |
| Female | 1,099 | 0,241 | 0,43 | 0,668 | [0,72-1,69] |
| Age | 1,062 | 0,009 | 7,16 | 0,000 | [1,05-1,08] |
| ADL functionality score | 1,715 | 0,191 | 4,86 | 0,000 | [1,38-2,13] |
| Dummy reablement user | 0,775 | 0,214 | -0,92 | 0,356 | [0,45-1,33] |

# OLS regression on long term care

Number of observations: 306

F: 8,70

Adjusted R^2^: 0,092

| **Variable** | **Coefficient** | **Std.error** | **T score** | **P>|z|** | **95% confidence interval** |
| --- | --- | --- | --- | --- | --- |
| Female | 0,101 | 0,041 | 2,45 | 0,015 | [0,02 – 0,18] |
| Age | 0,004 | 0,001 | 3,99 | 0,000 | [0,002 – 0,006] |
| ADL functionality score | 0,093 | 0,037 | 2,49 | 0,013 | [0,02 – 0,17] |
| Dummy reablement user | -0,057 | 0,038 | -1,51 | 0,132 | [-0,13 – 0,02] |
| Constant | -0,341 | 0,095 | -3,58 | 0,000 | [-0,53 – -,15] |

# Cox proportional hazard model on mortality

Number of subjects: 849

Number of failures: 167

Time at risk: 801 607

LR chi^2: 249,09

Log likelihood: -926,49

| **Variable** | **Hazard ratio** | **Std.error** | **Z score** | **P>|z|** | **95% confidence interval** |
| --- | --- | --- | --- | --- | --- |
| Female | 0,573 | 0,092 | -3,49 | 0,000 | [0,42-0,78] |
| Age | 1,072 | 0,007 | 10,54 | 0,000 | [1,06-1,09] |
| ADL functionality score | 1,538 | 0,129 | 5,12 | 0,000 | [1,30-1,81] |
| Dummy reablement user | 0,702 | 0,145 | -1,71 | 0,087 | [0,47-1,05] |

# OLS regression on mortality

Number of observations:306

F: 19,24

Adjusted R^2^: 0,193

| **Variable** | **Coefficient** | **Std.error** | **T score** | **P>|z|** | **95% confidence interval** |
| --- | --- | --- | --- | --- | --- |
| Female | 0,000 | 0,047 | 0,00 | 0,998 | [-0,09 – 0,09] |
| Age | 0,006 | 0,001 | 5,71 | 0,000 | [0,004 – 0,009] |
| ADL functionality score | 0,238 | 0,043 | 5,58 | 0,000 | [0,15 – 0,32] |
| Dummy reablement user | -0,081 | 0,044 | -1,86 | 0,063 | [-0,17 – 0,004] |
| Constant | -0,592 | 0,109 | -5,42 | 0,000 | [-0,81 – -0,38] |
